# SLFN12 Expression Significantly Effects the Response to Chemotherapy Drugs in Triple-Negative Breast Cancer

**DOI:** 10.3390/cancers16223848

**Published:** 2024-11-16

**Authors:** Savannah R. Brown, Emilie Erin Vomhof-DeKrey, Sarmad Al-Marsoummi, Trysten Beyer, Bo Lauckner, Mckenzie Samson, Sarah Sattar, Nicholas D. Brown, Marc D. Basson

**Affiliations:** 1 Department of Pathology, School of Medicine and the Health Sciences, University of North Dakota, Grand Forks, ND 58202, USA; savannah.corradi@und.edu (S.R.B.); emilie.dekrey@und.edu (E.E.V.-D.); 2 Department of Biomedical Sciences, School of Medicine and the Health Sciences, University of North Dakota, Grand Forks, ND 58202, USA; sarmad.al.marsoummi@und.edu (S.A.-M.); trysten.beyer@und.edu (T.B.); bo.h.lauckner@und.edu (B.L.); mckenzie.samson@und.edu (M.S.); sarah.sattar@ndus.edu (S.S.); nicholas.d.brown@und.edu (N.D.B.); 3 Department of Surgery, School of Medicine and the Health Sciences, University of North Dakota, Grand Forks, ND 58202, USA; 4 Department of Surgery, Northeast Ohio Medical University, Rootstown, OH 44272, USA; 5 University Hospitals-NEOMED, Cleveland, OH 44106, USA

**Keywords:** triple-negative breast cancer, SLFN12, chemotherapy, signature genes, IFN-α2

## Abstract

Background/Objectives: Schlafen12 (SLFN12) is an intermediate human Schlafen protein shown to correlate with survivability in triple-negative breast cancer (TNBC). SLFN12 causes differential expressions of significant cancer genes, but how they change in response to chemotherapy remains unknown. Our aim is to identify the effect of chemotherapy on genes that improve TNBC outcomes and other SLFN family members following SLFN12 knockout or overexpression. Methods: We overexpressed SLFN12 using a lentiviral vector and knocked out SLFN12 (AdvShSLFN12) using a hairpin adenovirus in MDA-MB-231 TNBC cells. Cells were treated with camptothecin, paclitaxel, zoledronic acid, or carboplatin to evaluate the SLFN12 signature cancer genes associated with improved TNBC outcomes using qPCR. Additionally, cells were treated alone and in combination with AdvShSLFN12, IFN-α2 (known SLFN12 stimulator), carboplatin, and paclitaxel. After treatment, the viable cell numbers were analyzed utilizing a colorimetric crystal violet assay for cell viability. Results: The SLFN family and SLFN12 cancer signature gene mRNA expressions were analyzed by RT-qPCR. Treating SLFN12-overexpressing TNBC cells with chemotherapy agents resulted in the differential expressions of eight cancer-related genes. Notably, GJB3 was downregulated following treatment with each chemotherapeutic drug. Inducing SLFN12 with IFN-α2 resulted in decreased cell viability and increased SLFN12 mRNA levels following treatment with paclitaxel or carboplatin. Conclusions: These results suggest that SLFN12 overexpression significantly affects the expressions of genes driving phenotypic changes in response to chemotherapy and influences additional SLFN family members following IFN-α2 treatment. This may contribute to improving the survival of patients with SLFN12 overexpression. Additionally, patient SLFN12 levels can be used as a factor when pursuing personalized chemotherapy treatments.

## Simple Summary

Triple-negative breast cancer (TNBC) is an aggressive breast cancer subtype, has poor prognosis, and lacks targeted therapies. Schlafen12 (SLFN12) is a protein linked to survival in TNBC. SLFN12 influences the expressions of important cancer genes and other SLFN family members, but it is unclear how these genes change following chemotherapy. This study indicates that SLFN12 overexpressing TNBC cells with chemotherapy agents resulted in the differential expressions of eight cancer genes. Further, when TNBC cells were treated with chemotherapy and hairpin adenovirus to knock down SLFN12, IFN-α2 treatment was able to increase SLFN family mRNA expression and decrease cell viability. Together, these results indicate the importance of examining SLFN family interactions with gene profiles in an effort to produce a targeted treatment approach for TNBC patients.

## 1. Introduction

Breast cancer is the most diagnosed cancer worldwide and the second leading cause of death for women, following lung cancer [1,2]. Although classified as a single disease, breast cancer is characterized phenotypically into multiple subtypes by hormone status and biological makeup [2,3,4]. Triple-negative breast cancer (TNBC) is the most aggressive form of breast cancer. TNBC is unresponsive to targeted therapies, such as endocrine therapy, due to the lack of estrogen receptor (ER), progesterone receptor (PR), and human epidermal growth factor receptor 2 (HER2) [2]. Therefore, non-specific treatments, such as surgery, radiation, and chemotherapy, remain the standard of care for these patients, but they often develop adverse side effects, such as hair loss, nausea, vomiting, infertility, and anemia [2,5,6]. Following treatment, many TNBC patients develop chemoresistance and radioresistance, a perfect storm created by the absence of hormonal receptors paired with an enriched CD44^+^/CD24^−^ breast cancer stem cell (BCSC) population [7,8,9,10]. Therefore, there is a crucial need for developing personalized, precise, and targeted therapies for TNBC patients.

Schlafens (SLFNs) are a novel set of proteins expressed in humans, rodents, and other mammals [11]. SLFNs are classified into three subgroups based on molecular structure and size, as follows: short SLFNs (MW: 37–42 kDa), intermediate SLFNs (MW: 58–68 kDa), and long SLFNs (MW: 100–104 kDa, including an extra C-terminal domain) [8,11,12,13,14]. Humans express six Schlafens that are categorized into the intermediate (SLFN12 and SLFN12-Like) and long (SLFN5, SLFN11, SLFN13, and SLFN14) subgroups and do not express the short subgroup [8,11,14,15]. SLFN12 is in the intermediate group and acts primarily in the cytosol, as it lacks the nuclear targeting sequence that is found in the long SLFN family [8,11,14,15]. SLFN12 stimulates differentiation in enterocytes and prostate cancer cells, and its overexpression inhibits prostate cancer, triple-negative breast cancer (TNBC), and lung adenocarcinoma cell proliferation [8,12,13,14]. TNBC and lung adenocarcinoma patients have longer survival when SLFN12 is expressed at higher levels than tumors expressing less SLFN12 [8,12]. TNBC cell sensitivity to radiation, carboplatin, paclitaxel, zoledronic acid, and camptothecin was greater when SLFN12 was overexpressed [16]. These data indicate that SLFN12 levels could be used to customize radio- or chemotherapy in patients with TNBC [16].

Interferon-alpha (IFN-α2) treatment has been shown to induce SLFN family expression in TNBC while simultaneously reducing cell viability [10]. Interestingly, when SLFN12 is knocked down following IFN-α2 signaling, a complex SLFN family intra-regulation cascade emerged [10]. These data indicate that SLFN5, SLFN12-Like protein, and SLFN14 are not controlled by SLFN12 during IFN-α2 signaling, whereas SLFN11 and SLFN13 may be influenced by SLFN12 during this process [10]. This work suggested that SLFN family proteins work together to promote ordered control of cell viability during IFN-α2 signaling in TNBC [10]. Further exploring this issue may help to develop a more targeted and personalized approach for TNBC patients.

Cancers frequently escape tumor suppressors, such as SLFN12, by developing downstream mutations that change the regulated expression of genes necessary for cancer cell immortality. We investigated a set of genes that were downstream and associated with SLFN12 in order to characterize the gene set’s relationship to the survival of TNBC patients [17]. Based on RNA-seq analysis on TNBC xenograft tumors that were established from empty vector (EV-control) or overexpressing SLFN12-encoding lentivirus (LV-SLFN12) in MDA-MB-231 cells, we analyzed the eight SLFN12 signature genes that were differentially expressed [17]. PAEP, GJA1, EEF1A3, and NQO1 were downregulated in LV-SLFN12 xenograft tumors compared to empty vector control (EV-SLFN12), while UCA1, FBP1, CALB2, and GJB3 were upregulated [17]. Kaplan–Meier curves for the relapse-free survival of SLFN12 signature genes indicated a higher survival probability for TNBC patients when SLFN12 was highly expressed [17]. Additionally, there is a significant difference in SLFN12 signature genes between African Americans and Caucasian Americans, which could indicate a course for targeted therapy that could increase SLFN12 expression, which would then further increase or decrease the SLFN12 signature genes in the African American population and lead to better breast cancer survival rates [17].

In this study, we hypothesize and demonstrate that SLFN12 significantly affects the expressions of genes driving phenotypic changes in response to chemotherapy drugs and changes the expressions of other SLFN family proteins in TNBC. MDA-MB-231 cells, a standard TNBC cell line, were treated with either EV-SLFN12, LV-SLFN12, or adenovirus short-hairpin SLFN12 (AdvShSLFN12). Additionally, the cells were treated with standard chemotherapy treatments, including carboplatin, paclitaxel, camptothecin (CPT), or zoledronic acid (ZA). SLFN family members were examined following SLFN12 knockout, IFN-α2 stimulation, and chemotherapy treatment alone or in combination.

## 2. Methods and Materials

### 2.1. Cells and Reagents

Cell lines were acquired from the American Tissue Culture Collection (ATCC, Manassas, VA, USA). The MDA-MB-231 cells and BT-549 cells were cultured in DMEM (Genesee Scientific, El Cajon, CA, USA), and the Hs-578T cells were cultured in RPMI (Genesee Scientific). All cell lines were supplemented with 10% fetal bovine serum (FBS) (Genesee Scientific), penicillin (10,000 units)/streptomycin (10 mg/1 mL) (ThermoFisher Scientific, Waltham, MA, USA), and grown in 5% CO_2_ at 37 °C. Crystal violet C-6158 was purchased from Sigma-Aldrich (Burlington, MA, USA). IFN-α2 was purchased from Biolegend (San Diego, CA, USA). Carboplatin (NSC 241240; Catalog No. S1215), paclitaxel (NSC 125973; Catalog No. S1150), zoledronic acid (ZOL 446; Catalog No. S1314), and camptothecin (CPT; Catalog No. S1288) were obtained from Selleck Chem (Houston, TX, USA). The primers are listed in Supplemental Table S1 and were from IDT (Coralville, IA, USA) or BioRad. Information regarding the chemotherapeutic drugs’ actions is listed in Supplemental Table S2.

### 2.2. Viral Constructs

MDA-MB-231 cells stably overexpressing SLFN12 (LV-SLFN12) or a control vector (EV-control) (generated as previously described [8]) were cultured in DMEM supplemented with 10% FBS at 37 °C and 5% CO_2_. A short hairpin RNA adenovector targeting SLFN12 (AdvShSLFN12) was obtained from Vector Biolab (Malvern, PA, USA, #shADV-223642). The control virus was constructed from the pAdeno vector with only the CMV promoter, as described previously [12].

### 2.3. RNA Isolation and qPCR

MDA-MB-231 cells were seeded into 6-well plates at a density of 200,000 cells per well and allowed to attach for 24 h. The cells were treated for 48 h with camptothecin (1.25–5.0 μM), zoledronic acid (30–50 μM), paclitaxel (20–60 μM), or carboplatin (60–120 μM). Additionally, IFN-α2 (5550 units) or AdvShSLFN12 (4000 VP) were added to the experimental conditions if necessary. The dose of each compound was selected based on previously generated dose–response curves [10,16]. RNA was isolated using QIAshredders, the RNeasy Mini kit, and the QIAcube from Qiagen (Germantown, MD, USA), cDNA was synthesized with SMARTScribe (Takara, San Jose, CA, USA), and qPCR was performed as previously described [10]. The expressions were calculated from the threshold cycle (Ct) values by 2^−ΔΔCt^ fold change using RPLP0 or B2M as the reference gene for all experiments, excluding paclitaxel, which used POLR2A as the reference gene for comparison of the SLFN family genes. The primer probe sequences used were previously published and are listed in Supplementary Table S1.

### 2.4. Cell Viability

Cell viability was measured by a colorimetric crystal violet-based assay that determines the percentage of viable cells while excluding dead cells, as previously described [10,16,17]. MDA-MB-231, BT-549, and Hs-578T cells were treated with adenoviral vectors at 4000 viral particles (VP)/cell, IFN-α2 at 5500 units, paclitaxel (5 nM), or carboplatin (40 uM), at varying combinations of the stated drugs. The dose–response curves of the MDA-MB-231, BT549, and Hs-578T cells tested with carboplatin and paclitaxel can be found in Supplemental Figures S1 and S2. The doses used in this study were selected based on Supplemental Figures S1 and S2 and previously published cytotoxicity data [16]. Additionally, IFN-α2 dose–response curves have been previously completed [10].

### 2.5. Correlation Curves and Statistical Analysis

Correlation curves and data tables were generated using the statistical software R version 4.2.2. Fold changes in the qPCR were compared between each of the genes using the function cor.test() from the ggpublr package. This generated adjusted R-squared values, degrees of freedom, correlation coefficients, and P-values for each of the genes being compared. Treatment groups that had R square values greater than 0.50 had their fold changes graphed against each other using the package ggplot2. Regression lines were also included, with 95% confidence interval bands.

The data were expressed as the means ± SEMs and analyzed by GraphPad prism v9 (San Diego, CA, USA) using one-way ANOVA followed by Šídák’s multiple comparisons test, unless stated otherwise. All experiments were repeated a minimum of three times.

## 3. Results

### 3.1. SLFN Family mRNA Expression Variably Increases Following Chemotherapy and IFN-α2 Treatment Paired with the Loss of SLFN12

It is well known that both carboplatin and paclitaxel reduce breast cancer cell viability [18,19,20]. IFN-α2 has been shown to induce SLFN family members, while simultaneously reducing cell viability in TNBC [10]. We have previously shown that AdvShSLFN12 is able to decrease SLFN12 expression at the mRNA and protein levels [10]. We then paired SLFN12 knockdown with IFN-α2 treatment and observed that SLFN5, SLFN12-Like, and SLFN14 mRNA expression increased, while IFN-α2-induced an increase in SLFN11 and SLFN13 protein expressions were blunted, indicating that the expressions of SLFN11 and SLFN13 could be controlled by SLFN12 [10]. Then, the simultaneous knockdown of SLFN5, SLFN12, SLFN12-Like, and SLFN14 resulted in only a slight recovery of the reduced viability [10]. As a next step, we sought to determine whether SLFN12 knockout with AdvShSLFN12 would block the reduction in cell viability seen when TNBC cells were treated with IFN-α2 in combination with commonly administered chemotherapeutics, such as carboplatin and paclitaxel, or whether changes in other SLFN family members would suffice to permit this effect. With this information, we hypothesized that treating TNBC with IFN-α2 + AdvShSLFN12 and chemotherapeutics would result in increased expressions of SLFN5, SLFN12-Like, and SLFN14 while still decreasing TNBC cell viability.

**Figure 1:**
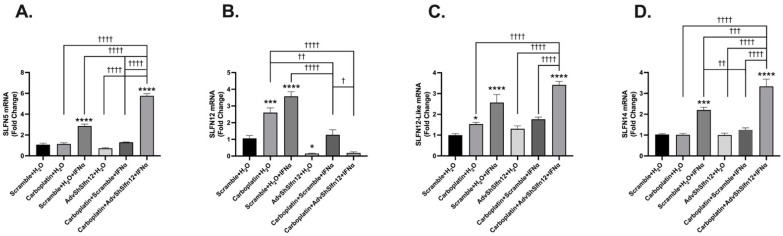
SLFN family mRNA expression variably increased following carboplatin treatment, paired with the loss of SLFN12 and IFN-α2 signaling. mRNA analysis performed by primer-probe RT-qPCR indicated that (**A**) SLFN5 (*n* = 6, *p* < 0.0001), (**C**) SLFN12-Like (*n* = 6, *p* < 0.0001), and (**D**) SLFN14 (*n* = 6, *p* < 0.0001) were induced by the IFN-α2 treatment and significantly further induced with the loss of SLFN12 and carboplatin in MDA-MB-231 cells. (**B**) Treatment with IFN-α2 was able to induce SLFN12, while AdvShSLFN12 or any paired treatment with AdvShSLFN12 decreased SLFN12 in MDA-MD-231 cells (*n* = 6, *p* < 0.0001). RPLP0 was used as the reference gene. All error bars shown represent the standard error of the mean. Asterisks denote a significant difference between the control and each condition, and crosses indicate significant differences among the shown conditions. The *p* value is for both asterisks and crosses; asterisks denote significant differences from the Scramble + H_2_O control, and crosses denote significant differences among the experimental groups.

Three TNBC cell lines, MDA-MB-231, BT-549, and HS-578T, were studied to ensure that the results were not specific to a single cell line. We evaluated the relationship of IFN-α2 and AdvShSLFN12 in TNBC with carboplatin or paclitaxel. Following treatment, SLFN5, SLFN12-Like, and SLFN14 mRNA levels were measured via qPCR to evaluate the relationships among SLFN family members. In the MDA-MB-231 cells treated with carboplatin alone, SLFN12 and SLFN12-Like had increased mRNA expression (Figure 1B,C, second bars) while SLFN5 and SLFN14 had no changes in their mRNA levels (Figure 1A,D, second bars). When stimulated with IFN-α2, each SLFN family member examined significantly increased mRNA expression (Figure 1A–D, third bars). When SLFN12 was knocked out (AdvShSlfn12+DMSO), there were no changes in SLFN5, SLFN12-Like, or SLFN14 expression compared to Scramble + H2O control (Figure 1A,C,D, fourth bars). SLFN5, SLFN12-Like, and SLFN14 each had the largest increase in mRNA expression following the treatment combination of carboplatin, IFN-α2, and AdvShSLFN12 (Figure 1A,C,D, sixth bars). Similar results were demonstrated in both the BT549 and Hs-578T cell lines, which can be found in Supplemental Figures S3 and S5 (“*” or “†” = *p* < 0.05; “**” or “††” = *p* < 0.01; “***” or “†††” = *p* < 0.001; “****” or “††††” = *p* < 0.0001, same as below).

**Figure 2:**
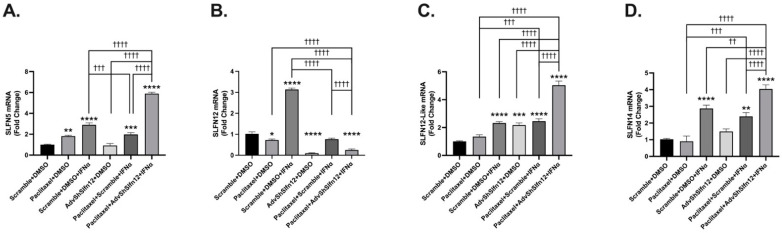
SLFN family mRNA expression variably increased following paclitaxel treatment, paired with the loss of SLFN12 and IFN-α2 signaling. mRNA analysis performed by primer-probe RT-qPCR revealed that (**A**) SLFN5 (*n* = 6, *p* < 0.0001), (**C**) SLFN12-Like (*n* = 6, *p* < 0.0001), and (**D**) SLFN14 (*n* = 6, *p* < 0.0001) were induced by IFN-α2 treatment and significantly further induced with the loss of SLFN12 and paclitaxel treatment in MDA-MB-231 cells. (**B**) Treatment with IFN-α2 was able to induce SLFN12 but not in the presence of AdvShSLFN12 or any paired treatment with AdvShSLFN12 in MDA-MD-231 cells (*n* = 6, *p* < 0.0001). POLR2A was used as the reference gene. All error bars shown represent the standard error of the mean. Asterisks denote a significant difference between the control and each condition, and crosses indicate significant differences among the shown conditions. The *p* value is for both asterisks and crosses; asterisks denote significant differences from the Scramble + H_2_O control, and crosses denote significant differences among the experimental groups.

Similarly, when TNBC cells were treated with paclitaxel alone, SLFN5 mRNA expression increased significantly, while SLFN12 expression was significantly decreased compared to the Scramble + DMSO control (Figure 2, second bar). Furthermore, SLFN12-Like and SLFN14 mRNA levels did not change compared to the Scramble + DMSO control (Figure 2, second bar). SLFN5, SLFN12, SLFN12-Like, and SLFN14 mRNA were each elevated following IFN-α2 stimulation (Figure 2, third bars). Only SLFN12-Like mRNA significantly increased after treatment with AdvShSLFN12 alone (Figure 2C, fourth bar). SLFN5, SLFN12-Like, and SLFN14 mRNA expression each increased following treatment with the combination of carboplatin or paclitaxel, IFN-α2, and AdvShSLFN12, suggesting that the loss of SLFN12 during IFN-α2 and chemotherapy treatment may have an additive effect on inducing the mRNA expression corresponding to these other proteins (Figure 2A,C,D, sixth bars). Similar results were demonstrated in both the BT549 and Hs-578T cell lines, which can be found in Supplemental Figures S4 and S6.

### 3.2. Chemotherapy, IFN-α2, and the Loss of SLFN12 Effectively Decreased TNBC Cell Viability

**Figure 3:**
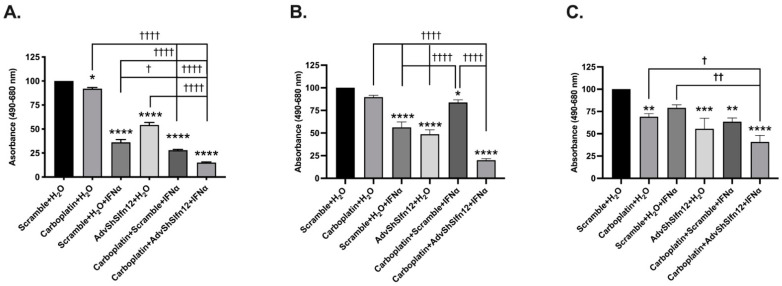
Carboplatin, IFN-α2, and the loss of SLFN12 effectively decreased TNBC cell viability. Crystal violet assay exhibited a decrease in cell viability following treatment with carboplatin, IFN-α2, or AdvShSLFN12, with the furthest decrease observed when all three treatments were combined in (**A**) MDA-MB-231 (*n* = 6, *p* < 0.0001), (**B**) BT-549 (*n* = 6, *p* < 0.0001), and (**C**) Hs-578T (*n* = 4, *p* = 0.0001). All error bars shown represent the standard error of the mean. Asterisks denote a significant difference between the control and each condition, and crosses indicate significant difference among the shown conditions. The *p* value is for both asterisks and crosses; asterisks denote a significant difference from the Scramble + H_2_O control, and crosses denote significant differences among the experimental groups.

**Figure 4:**
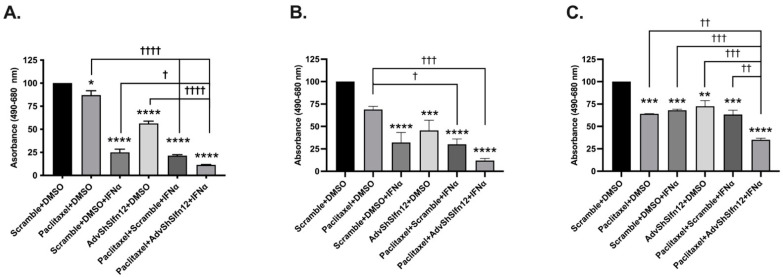
Paclitaxel, IFN-α2, and the loss of SLFN12 effectively decreased TNBC cell viability. Cell viability was measured with a crystal violet assay following treatment with paclitaxel, IFN-α2, or AdvShSLFN12, with the furthest decrease observed when all three treatments were combined in (**A**) MDA-MB-231 (*n* = 6, *p* < 0.0001), (**B**) BT-549 (*n* = 6, *p* < 0.0001), and (**C**) Hs-578T (*n* = 4, *p* = 0.0001). All error bars shown represent the standard error of the mean. Asterisks denote a significant difference between the control and each condition, and crosses indicate significant differences among the shown conditions. The *p* value is for both asterisks and crosses; asterisks denote a significant difference from the Scramble + H_2_O control, and crosses denote significant differences among the experimental groups.

Although we previously demonstrated that cell viability decreased with the loss of SLFN12 and IFN-α2 stimulation in TNBC [10], it remained unclear whether viability is further reduced by chemotherapeutic agents. In all three cell lines, treatment with carboplatin (second bars), IFN-α2, (third bars), or AdvShSLFN12 (fourth bars) decreased cell viability (Figure 3A–C). The combination of carboplatin with IFN-α2 reduced cell viability, but the combination of carboplatin, IFN-α2, and AdvShSLFN12 (sixth bars) resulted in the greatest reduction in viability across each cell line (Figure 3). Treatment with paclitaxel resulted in a similar pattern of cell viability reduction (Figure 4).

### 3.3. SLFN12 Signature Gene Response to Chemotherapy Agents

Previous work from our laboratory identified eight SLFN12 signature genes in TNBC whose expression is substantially altered after SLFN12 induction [17]. To determine whether chemotherapeutic agents affect SLFN12 gene signature expression in the MDA-MB-231 TNBC cell line, the cells were treated with the empty vector lentivirus control (EV-control) or overexpressing SLFN12 lentivirus (LV-SLFN12) in combination with chemotherapeutics. Previous work from our laboratory confirmed the overexpression of SLFN12 using LV-SLFN12 for both mRNA and protein expression [8,21]. Utilizing qPCR, the mRNA expression of each SLFN12 signature gene is evaluated in the following sections based on each chemotherapy drug used. Additional information on each signature gene is listed in Supplemental Tables S3 and S4.

#### 3.3.1. Camptothecin

**Figure 5:**
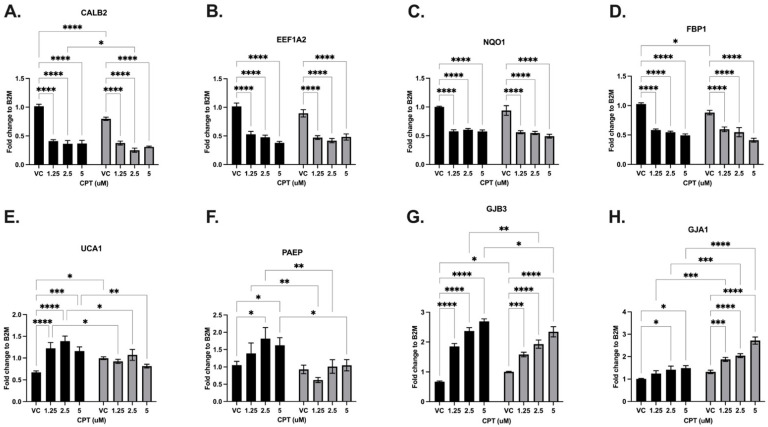
Camptothecin variably changed the SLFN12 signature cancer gene response. mRNA analysis was carried out with primer-probe RT-qPCR using increasing concentrations of CPT treatment and EV-control (black bars) or LV-SLFN12 (grey bars) for (**A**) CALB2 (*n* = 5, *p* < 0.0001), (**B**) EEF1A2 (*n* = 5, *p* < 0.0001), (**C**) NQO1 (*n* = 4, *p* < 0.0001), (**D**) FBP1 (*n* = 4, *p* < 0.0001), (**E**) UCA1 (*n* = 5, *p* < 0.0001), (**F**) PAEP (*n* = 5, *p* < 0.0001), (**G**) GJB3 (*n* = 4, *p* < 0.0001), and (**H**) GJA1 (*n* = 5, *p* < 0.0001). B2M was used as the reference gene. All error bars shown represent the standard error of the mean.

MDA-MB-231 EV-control and LV-SLFN12 cells were treated with camptothecin (CPT) at concentrations of 0, 1.25, 2.50, and 5.00 μM. The CPT chemotherapy resulted in decreased expressions of CALB2, EEF1A2, NQO1, and FBP1, while UCA1, PAEP, GJB3 and GJA1 had increased mRNA expressions at one or more of the CPT dose concentrations in comparison to the vehicle control (VC) (Figure 5, black bars). The mRNA expressions of CALB2, FBP1, UCA1, and GJB3 decreased in the VC LV-SLFN12 compared to in the VC EV-control (Figure 5A,D,E,G). The mRNA expressions of EEF1A2, NQO1, PAEP, and GJA1 were not affected by the overexpression of SLFN12 (Figure 5B,C,F,H). At one or more CPT doses with LV-SLFN12, there were greater decreased mRNA expressions of CALB2 and PAEP (Figure 5A,F). There were significant decreases in the CPT-induced expressions of UCA1 and GJB3 due to the increased SLFN12 expression, whereas the expression of GJA1 was heightened further in the CPT + LV-SLFN12 samples (Figure 5E,G,H).

#### 3.3.2. Zoledronic Acid

**Figure 6:**
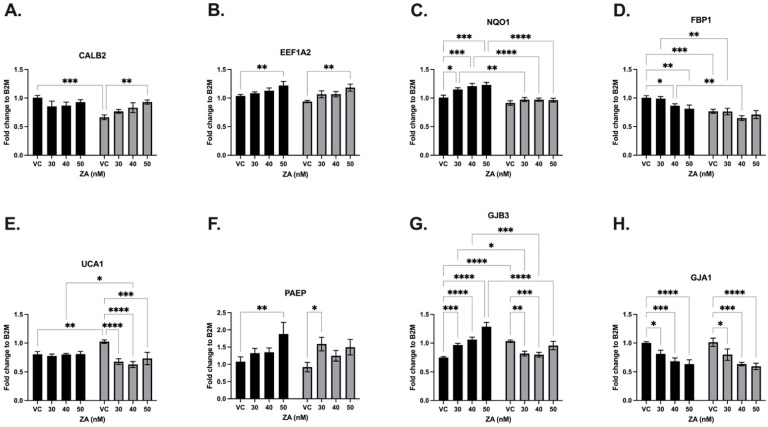
Zoledronic acid variably changed the SLFN12 signature cancer gene response. mRNA analysis was carried out with primer-probe RT-qPCR using increasing concentrations of ZA treatment and EV-control (black bars) or LV-SLFN12 (grey bars) for (**A**) CALB2 (*n* = 5, *p* < 0.0001), (**B**) EEF1A2 (*n* = 5, *p* < 0.0001), (**C**) NQO1 (*n* = 4, *p* < 0.0001), (**D**) FBP1 (*n* = 4, *p* < 0.0001), (**E**) UCA1 (*n* = 5, *p* < 0.0001), (**F**) PAEP (*n* = 5, *p* < 0.0001), (**G**) GJB3 (*n* = 4, *p* < 0.0001), and (**H**) GJA1 (*n* = 5, *p* < 0.0001). B2M was used as the reference gene. All error bars shown represent the standard error of the mean.

MDA-MB-231 cells were treated with zoledronic acid (ZA) at concentrations of 0, 30, 40, and 50 μM in the EV-SLFN12 and LV-SLFN12 groups. Treatment with ZA resulted in decreased expressions of FBP1, UCA1 and GJA1, whereas increased mRNA expressions were observed for EEF1A2, NQO1, PAEP, and GJB3 (Figure 6, black bars). When comparing the LV-SLFN12 and EV-control groups, CALB2, FBP1, UCA1, and GJB3 each had significantly decreased mRNA expression (Figure 6A,D,E,G). When the cells were treated with ZA, the mRNA expressions of NQO1, FBP1, UCA1, and GJB3 were significantly downregulated due to the increase in SLFN12 (Figure 6C,D,E,G). Conversely, SLFN12 overexpression did not alter the ZA-induced changes in the mRNA expressions of CALB2, EEF1A2, UCA1, PAEP, or GJA1 (Figure 6A,B,E,F,H).

#### 3.3.3. Paclitaxel

**Figure 7:**
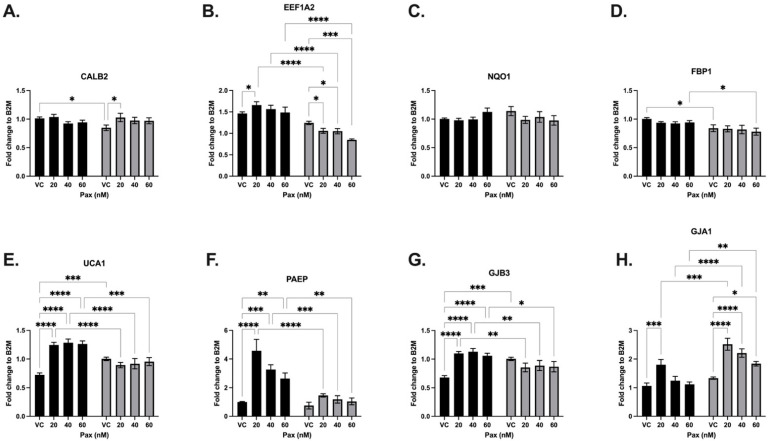
Paclitaxel variably changed the SLFN12 signature cancer gene response. mRNA analysis was carried out with primer-probe RT-qPCR using increasing concentrations of paclitaxel treatment and EV-control (black bars) or LV-SLFN12 (grey bars) for (**A**) CALB2 (*n* = 5, *p* < 0.0001) (**B**) EEF1A2 (*n* = 5, *p* < 0.0001), (**C**) NQO1 (*n* = 4, *p* < 0.0001), (**D**) FBP1 (*n* = 4, *p* < 0.0001), (**E**) UCA1 (*n* = 5, *p* < 0.0001), (**F**) PAEP (*n* = 5, *p* < 0.0001), (**G**) GJB3 (*n* = 4, *p* < 0.0001), and (**H**) GJA1 (*n* = 5, *p* < 0.0001). B2M was used as the reference gene. All error bars shown represent the standard error of the mean.

MDA-MB-231 EV-control and LV-SLFN12 cells were treated with paclitaxel (Pax) at 0, 20, 40, or 60 μM. Paclitaxel increased the expressions of EEF1A2, UCA1, PAEP, and GJA1 (Figure 7B,E,F,H, black bars). When comparing the VC LV-SLFN12 and VC EV-control groups, CALB2, FBP1, UCA1, and GJB3 each had significantly decreased mRNA expressions (Figure 7A,D,E,G). EEF1A2, FBP1, UCA1, PAEP, and GJB3 were significantly downregulated when SLFN12 was overexpressed at one or more of the paclitaxel dose concentrations (Figure 7B,D–G). Conversely, Pax plus LV-SLFN12 resulted in a greater increased mRNA expression of GJA1 (Figure 7H) but no changes in CALB2 or NQO1 (Figure 7A,C).

#### 3.3.4. Carboplatin

**Figure 8:**
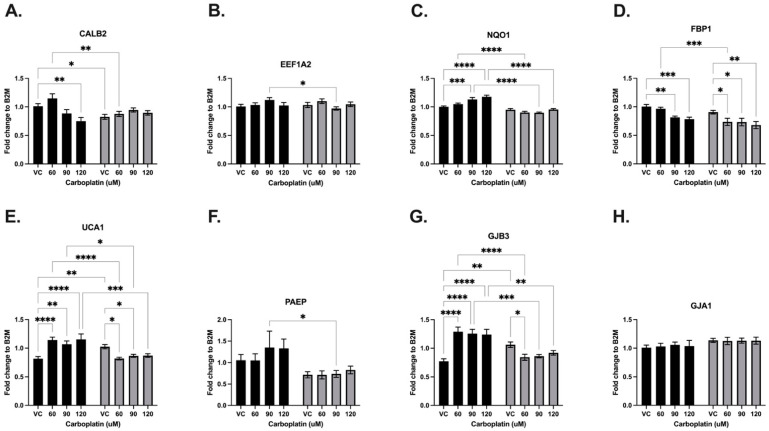
Carboplatin variably changed the SLFN12 signature cancer gene response. Increasing concentrations of carboplatin treatment were analyzed by mRNA primer-probe RT-qPCR with EV-control (black bars) or LV-SLFN12 (grey bars) for (**A**) CALB2 (*n* = 5, *p* < 0.0001), (**B**) EEF1A2 (*n* = 5, *p* < 0.0001), (**C**) NQO1 (*n* = 4, *p* < 0.0001), (**D**) FBP1 (*n* = 4, *p* < 0.0001), (**E**) UCA1 (*n* = 5, *p* < 0.0001), (**F**) PAEP (*n* = 5, *p* < 0.0001), (**G**) GJB3 (*n* = 4, *p* < 0.0001), and (**H**) GJA1 (*n* = 5, *p* < 0.0001). B2M was used as the reference gene. All error bars shown represent the standard error of the mean.

Carboplatin was administered at concentrations of 0, 60, 90, and 120 μM to EV-control and LV-SLFN12 MDA-MB-231 cells. The carboplatin treatment resulted in significantly decreased mRNA expressions of CALB2 and FBP1, while significantly increased mRNA expressions were observed for NQO1 and GJB3 (Figure 8, black bars). When comparing the VC LV-SLFN12 and VC EV-control groups, CALB2, FBP1, UCA1, and GJB3 each had significantly decreased mRNA expressions (Figure 8A,D,E,G). SLFN12 overexpression caused a significant decreased mRNA expression for CALB2, EEF1A2, NQO1, FBP1, UCA1, PAEP, and GJB3 at one or more concentrations of carboplatin plus LV-SLFN12 (Figure 8A–G). GJA1 was not affected by SLFN12 overexpression or carboplatin treatment (Figure 8H).

#### 3.3.5. Correlative Effects of Chemotherapy Agents and SLFN12 Overexpression

**Figure 9:**
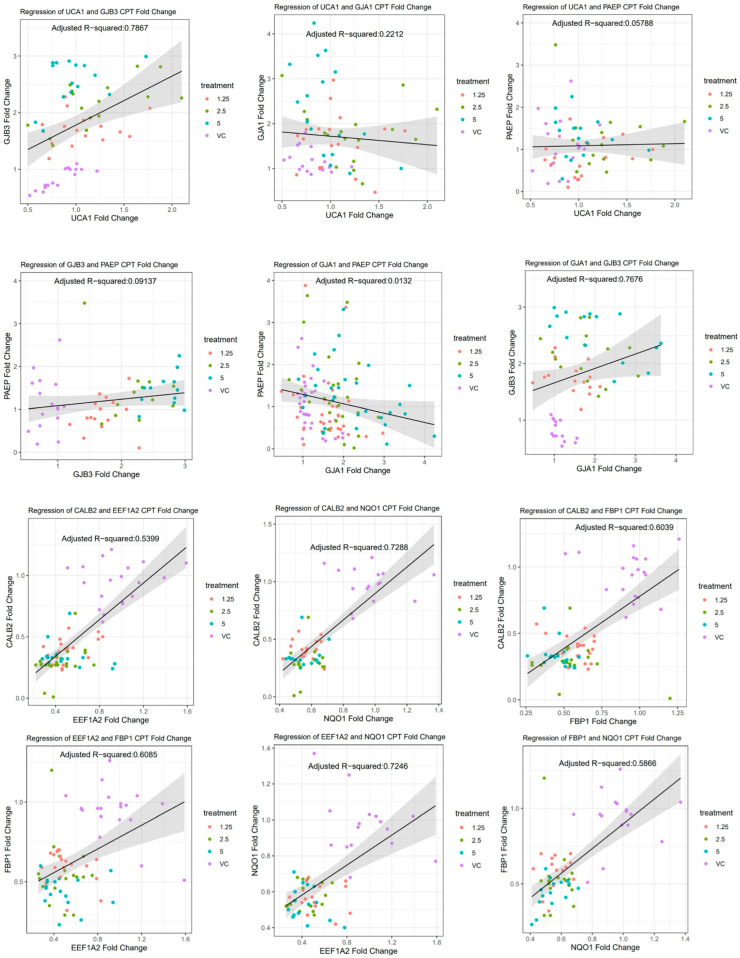
Correlative effects of chemotherapy agents and SLFN12 overexpression. Statistical software R version 4.2.2 was utilized to compare the fold changes in the SLFN12 signature genes using the function cor.test() from the ggpublr package, which were then plotted with the ggplot2 package. Regression lines were included with 95% confidence interval bands.

Since it appears that some of the chemotherapy agents resulted in similar up- or downregulation of the SLFN12 signature genes, we analyzed the correlations of the signature genes that changed in their mRNA expressions similarly to each other within each chemotherapy treatment (Supplementary Table S5). The R^2^ values display the strongest relationship correlation for the SLFN12 signature genes with the CPT treatment (Figure 9). The correlation data tables for carboplatin, ZA, and paclitaxel did not have strong R^2^ values (Supplementary Table S5).

## 4. Discussion

TNBC tumors are unresponsive to targeted therapy, including endocrine therapy, and are often chemoresistant. Therefore, there is an immediate need for new approaches to treating this disease. SLFN12 correlates with improved survival in TNBC patients and has been shown to increase sensitivity to both chemotherapy and radiation treatments [8,10,16]. In recent studies, IFN-α2 has been shown to stimulate SLFN gene expression and acts as the only known stimulator for SLFN12 [10]. Our present study suggests that the combination of IFN-α2 and chemotherapy reduces TNBC cell viability while increasing SLFN5, SLFN12-Like, and SLFN14 mRNA expression in the absence of SLFN12. Furthermore, treatment with chemotherapy in SLFN12-overexpressing TNBC cells resulted in differential responses in SLFN12 signature cancer genes—providing further insight into targeted treatment options for TNBC patients, as demonstrated in Table 1. Taken together, these data suggest that the overexpression of SLFN12 impacts the expression of genes driving phenotypic changes in response to chemotherapy, which affects additional SLFN family members following IFN-α2 treatment. Understanding this relationship may enhance the survival of patients with elevated SLFN12 expression. Furthermore, patient SLFN12 levels may serve as a promising factor when pursuing personalized chemotherapy treatments.

While interferons have been known for over 50 years to cause anti-tumor effects [22], IFN-α2 has recently been shown to influence the expressions of SLFN family members. However, how SLFNs contribute to the activity of interferons remains generally ambiguous [11,15,23]. IFN-α2 induces complex interactions among SLFN family members [10], but how chemotherapeutic agents might affect these interactions has not been explored. Our current study indicates that the loss of SLFN12 during IFN-α2 and exposure to chemotherapeutic agents may have an additive effect on TNBC cells, increasing SLFN family mRNA levels and decreasing cell viability. This study may be particularly relevant for TNBC tumors with lower levels of SLFN12. In such tumors, IFN-α2 treatment could induce SLFN family members in order to decrease TNBC cell viability. While IFN-α2 treatment remains the only viable option for SLFN12 stimulation, further studies would be required to move to a clinical-based setting, as its administration differs between our in vitro setting (5500 IU/mL) compared to cancer therapy treatments at (9 million U subcutaneously) up to three times a week [10,24,25]. SLFN12 sensitizes TNBC cells to both chemotherapy drugs and radiotherapy [16]. Additionally, they showed that overexpressing SLFN12 TNBC cells resulted in sensitization to carboplatin, paclitaxel, ZA, CPT, and cesium irradiation compared to the control TNBC cell population [16]. Our current study seems to reflect this pattern by decreasing cell viability levels following carboplatin or paclitaxel treatment in SLFN12-induced IFN-α2 stimulated cells.

Our results also suggest that determining SLFN12 signature gene expression patterns following SLFN12 overexpression and chemotherapy treatment may play an essential role in understanding TNBC pathogenesis. GJB3, a member of the connexin gene family, was shown in previous work to be upregulated when SLFN12 was overexpressed [17] but when LV-SLFN12 TNBC cells were treated with each chemotherapy, the GJB3 expression was downregulated (summarized in Table 1). In a recent comprehensive pan-cancer prognosis review, GJB3 survival analysis indicated that breast invasive carcinoma (BRCA) patients with higher GJB3 levels had a longer overall survival time compared to BRCA patients with lower levels of GJB3 [26]. Additionally, when the tumor clinicopathological state was evaluated, advanced BRCA patients (stage III and IV) showed significantly lower levels of GJB3—collectively suggesting that GJB3 promotes tumor-suppressive characteristics and may serve as a potential biomarker for both the detection and staging of breast cancer [26]. Zeng et al. study also revealed GJB3-resistant drugs, RO-3316 and cisplatin, which primarily target the PI3L/MTOR and cell cycle signaling pathways [24]. Synergistic effects on cancer growth via enhanced connexin expression have been described in numerous studies [27,28,29,30,31,32], yet the role for each connexin protein appears to be specific and must be carefully assessed to avoid a detrimental clinical outcome. For example, the upregulation of GJA1, or Cx43, sensitizes colorectal cancer cells to doxorubicin, florouracil, oxaliplatin, and taxol-based treatments [27,30,33]. Interestingly, we observed an increase in GJA1 following CPT and paclitaxel treatment but not after carboplatin or ZA treatment, indicating the need for specific assessment of each connexin in personalized cancer treatment. Cx32 inhibition also modulates cisplatin resistance in ovarian cancer cells [26,34]. Taken together, these data can provide insight into the regulation patterns observed following chemotherapy treatment in LV-SLFN12 TNBC cells.

Previously, SLFN12 overexpression was reported to regulate long non-coding RNA UCA1 [17]. We observed that treating SLFN12-overexpressing TNBC with CPT, paclitaxel, or carboplatin reduced UCA1 gene expression. UCA1 is regulated by the activation of hypoxia-inducible transcription factor (HIF-1) and has been implicated in the promotion of development and proliferation of breast cancer cells through numerous pathways by regulating cell-cycle proliferation and tumor metastasis and apoptosis [35,36,37,38,39]. Interestingly, UCA1 was upregulated in MDA-MB-231 TNBC cells compared to matched normal tissues under hypoxic conditions, suggesting that increased UCA1 expression may contribute to the increased migration and invasion of hypoxic breast cancer cells [34]. In our studies, we observed that UCA1 was upregulated following the overexpression of SLFN12, but UCA1 became downregulated following treatment with carboplatin and paclitaxel, which inhibit cell cycle progression, and CPT, which induces apoptosis via the inhibition of topoisomerase I. Taken together, understanding SLFN12 signature genes along with their expressions following chemotherapy treatment may provide a comprehensive understanding for personalized chemotherapy treatments in the future.

Our previous observations indicated that the effects of IFN-α2 on TNBC are not purely mediated through SLFN12 but rather through the SLFN family members that influence each other’s expressions in a complex fashion [10]. Likewise, our current study indicates that knocking out SLFN12 in TNBC and simultaneously treating it with IFN-α2 and carboplatin or paclitaxel increases the expressions of SLFN5, SLFN12-Like, and SLFN14 while diminishing TNBC cell viability. While chemotherapy alone reduced cell viability, the mitogenic effect of IFN-α2 inducing other SLFN family member expressions may contribute to the greater decrease observed in TNBC cell viability following IFN-α2 treatment; however, how each SLFN family member influences the other remains vague [8,10,16,22]. SLFN11 has been highlighted as a potential marker for cancer cell chemosensitivity in platinum-based drugs [40], topoisomerase inhibitors [41,42,43,44,45], poly-ADP ribose inhibitors [46,47,48,49,50,51], and antibody-drug conjugates [45] in over ten cancer types [11]. Furthermore, SLFN12 levels have been shown to not only affect intrinsic TNBC tumor biology but also the response to treatment by sensitizing TNBC to radiation and cytotoxic drugs [16]. It has been proposed that SLFN12 increases the sensitivity by reducing the phosphorylation of CHK1 and CHK2 [16]. Together, these data suggest that SLFN family members may require a balance in their expression levels to maintain or decrease cell viability.

While this study primarily focused on the role of SLFN12 along with SLFN5, SLFN12-Like, and SLFN14, we must address SLFN11 and SLFN13. In our previous study [10], SLFN family members’ mRNA expressions indicated that SLFN5, SLFN12-Like, and SLFN14 are not controlled by SLFN12 during IFN-α2 signaling. SLFN12 may be necessary for SLFN11 and SLFN13 induction during IFN-α2 signaling, as SLFN12 knockdown did lead to a decrease in SLFN11 or SLFN13 mRNA and protein expression. Since SLFN5, SLFN12-Like, and SLFN14 were not controlled by SLFN12 during IFN-α2 signaling, TNBC cell viability was investigated for these SLFN family members. The TNBC cell viability analysis revealed a novel intra-regulated signaling cascade during IFN-α2 signaling among SLFN family members, which indicated ordered control of cell viability among the SLFN family members, where the control is from highest to lowest as follows: SLFN14 > SLFN12-Like > SLFN5 > SLFN12 [10]. We did not explore SLFN11 and SLFN13 involvement in TNBC cell viability since it appeared that their expressions were controlled by SLFN12; however, that does not take away from their roles as investigated by others in cancer biology [11,16,52]. SLFN11 has been shown to sensitize cancer cells to chemotherapy and radiation through five different mechanisms, which may allow for SLFN11 to act as a biomarker for predicting a patient’s response to DNA-damaging agents [11,16,52]. SLFN13 has been shown to be downregulated in breast cancer, lung squamous carcinoma, prostate cancer, and rectal carcinoma [11]. In contrast, these proteins are all upregulated in CNS tumors, pancreatic, and renal cell carcinoma [11]. Further exploration into the roles of SLFN11 and SLFN13 needs be carried out in the future for TNBC.

Moreover, SLFN proteins play important roles in the regulation of immune responses and cellular processes [11,23]. As an RNase (ribonuclease), SLFN12 can degrade RNA molecules, which may help modulate gene expression and immune signaling by eliminating unwanted or viral RNAs [53,54,55]. Key findings from Garvie et al. indicate that the binding of PDE3A stabilizes SLFN12, promoting its transition into an active RNase form [53]. These findings further suggest that SLFN12 activation is linked to facilitating an immune response through targeting viral RNAs, which may provide new strategies for manipulating SLFN12 for therapeutic applications [53]. As a helicase, SLFN12 is involved in unwinding RNA and DNA, facilitating processes such as replication and transcription [11,53,55]. Together, the RNase and helicase functions contribute to the maintenance of cellular homeostasis and the response to stressors, highlighting the intricate connections between RNA metabolism and immune regulation. In the future, exploration into mutations within the active site of SLFN12 would be interesting to see if a similar effect occurs on SLFN family expressions and changes in SLFN12 signature cancer genes.

## 5. Conclusions

Taken together, with previous studies demonstrating the complex relationships among SLFN family members [10] and the discovery of SLFN12 signature cancer genes [17], these results indicate the importance of examining SLFN family interactions along with gene profiles in order to define a targeted treatment approach for TNBC patients. SLFN5, SLFN12-Like, and SLFN14 mRNA expressions variably increase following exposure to chemotherapeutic agents paired with the loss of SLFN12 and IFN-α2 signaling. Furthermore, this treatment combination was able to effectively decrease TNBC cell viability. Significant decreases in the chemotherapy-induced expressions of numerous SLFN12 signature cancer genes were observed following SLFN12 overexpression, highlighting the importance of understanding the comprehensive makeup of the SLFN12 signature cancer genes to more accurately develop a personalized treatment plan for TNBC patients. Overall, these data indicate the importance of understanding the role of SLFN12 in TNBC, which may contribute to improving survival for patients with increased or decreased levels of SLFN12. Furthermore, patient SLFN12 levels may be used as a factor when pursuing targeted chemotherapy treatments in the future.

## Figures and Tables

**Table 1:** LV-SLFN12 expression before and after chemotherapy treatment.

**A**									
	**CALB2**	**EEF1A2**	**NQO1**		**FBP1**	**UCA1**	**PAEP**	**GJB3**	**GJA1**
**LV-SLFN12**									
**B**									
	**CALB2**	**EEF1A2**	**NQO1**		**FBP1**	**UCA1**	**PAEP**	**GJB3**	**GJA1**
**CPT**									
**ZA**									
**Paclitaxel**									
**Carboplatin**									
	**Upregulated**			**Downregulated**			**No Change**		
**KEY**									

(A) Previous work [17] demonstrated that when SLFN12 was overexpressed with LV-SLFN12, the mRNA expressions of CALB2, FBP1, UCA1, and GJB3 were upregulated, while those of EEF1A2, NQO1, PAEP, and GJA1 were downregulated. Panel (B) highlights the SLFN12 signature gene expressions with LV-SLFN12 and chemotherapy treatment. CPT treatment resulted in the upregulation of GJA1, the downregulation of UCA1, PAEP, and GJB3, and no changes in the mRNA expressions of CALB2, EEF1A2, NQO1, or FBP1. ZA treatment downregulated NQO1, FBP1, and GJB3 and resulted in no changes in the expressions of CALB2, EEF1A2, UCA1, PAEP, or GJA1. Paclitaxel upregulated EEF1A2 and GJA1, downregulated FBP1, UCA1, PAEP, and GJB3, and did not affect the CALB2 and NQO1 mRNA expressions. Carboplatin treatment decreased CALB2, NQO1, UCA1, and GJB3 and had no effects on the EEF1A2, FBP1, PAEP, or GJA1 mRNA expressions.

## Data Availability

The supporting data from this study will be made available upon request.

## Supplementary Materials

The following supporting information can be downloaded at https://www.mdpi.com/article/10.3390/cancers16223848/s1, Figure S1: Carboplatin dose–response in TNBC, Figure S2: Paclitaxel dose–response in TNBC, Figure S3: SLFN family mRNA expression variably increased following carboplatin treatment paired with the loss of SLFN12 and IFN-α2 signaling in BT-549 cells, Figure S4: SLFN family mRNA expression variably increased following carboplatin treatment paired with the loss of SLFN12 and IFN-α2 signaling in Hs-578T cells, Figure S5: SLFN family mRNA expression variably increased following paclitaxel treatment paired with the loss of SLFN12 and IFN-α2 signaling, Figure S6: SLFN family mRNA expression variably increased following paclitaxel treatment paired with the loss of SLFN12 and IFN-α2 signaling in Hs-578T cells; Table S1: Primers, Table S2: Chemotherapy information, Table S3: SLFN12 upregulated signature genes, Table S4: SLFN12 upregulated signature genes, Table S5: Correlation curve analysis.
