# Supplementary material for: SLFN12 Expression Significantly Effects the Response to Chemotherapy Drugs in Triple-Negative Breast Cancer
Source: Cancers (Basel). 2024 Nov 16;16(22):3848. doi: 10.3390/cancers16223848 (PMC11593201; doi:10.3390/cancers16223848)
Supplement: Supplementary file 1 [file cancers-16-03848-s001.zip › cancers-3281411-supplementary.pdf]

Gene	Forward	Reverse	Probe
Human RPLP0	qHsaCEP0041375		
Human B2M	5’-CCACTGAAAAAGATGAGTATGCCT-3’	5’-CCAATCCAAATGCGGCATCTTCA-3’	5HEX/CCTGCCGTG/ZEN/TGAACCATGTGACT/3IABkFQ/
Human POLR2A	CAGTTCGGAGTCCTGAGTC	TCGTCTCTGGGTATTTGATGC	/5HEX/ACTGAAGCG/ZEN/AATGTCTGTGACGGAG /3IABkFQ/
Human SLFN5	5'-TGGCAGATGACAAACTCAACT-3'	5'-GAGAATGGACTGCTTGGATGA-3'	5'-/56-FAM/CCTGGAAAG/ZEN/GTCTGGGTCAGCTT/3IABkFQ/-3'
Human SLFN12	5’- GGGAGCAGGTAATGACGTATTTATT- 3’	5’- CAGTTGACCAGGAAGGAATGG-3’	5’-/56-FAM/ATCCAGTTC/ZEN/ ATGGTGGAGGCTGAA/3IABkFQ/−3’
Human SLFN12-Like	5'-GCTCAGCATAGTTTGTGTCTAA-3'	5'-ATGGACCTCGCCAGAAA-3'	5'-/56-FAM/TGGAAATGG/ZEN/CTTAGCTGCTGGGAA/3IABKFQ/-3'
Human SLFN14	qHsaCEP0056002		

Supplemental Table S1. Primers

B2M, POLR2A, SLFN5, SLFN12, and SLFN12-Like were obtained from Integrated DNA Technology (IDT). SLFN14 and RPLP0 primers were obtained from BioRad.

Inhibitor	Dose	Target	Function
Camptothecin (CPT)	1.25 μM – 5.00 μM	Topoisomerase I (Topo I)	Inhibit tumor cell proliferation and induce apoptosis via inhibiting Topo I
Zoledronic Acid (ZA)	30 μM – 50 μM	Bisphosphonate	Lowers high blood calcium levels via reducing the amount of calcium released from bone to blood.
Paclitaxel	20 μM – 60 μM	Microtubules	Promote assembly of microtubules from tubulin dimers. Stabilizes microtubules by preventing depolymerization.
Carboplatin	60 μM – 120 μM	DNA Replication	Reactive platinum complexes allow for cross-linkage of DNA molecules within the cell – leading to DNA strand breakage during replication.

Supplemental Table S2. Chemotherapy Information

Gene	Function	Regulation Following LV-SLFN12
CALB2	Calbindin 2<br><br>Calcium binding protein primarily expressed by cells in the nervous system and ovarian cells. Thought to modulate apoptosis downstream of calcium release, as when it is downregulated cells are much less likely to undergo apoptosis.	Upregulated
FBP1	Fructose-Bisphosphatase 1<br><br>Gluconeogenesis regulatory enzyme which catalyzes the hydrolysis of fructose 1,6-bisphosphate to fructose 6-phosphate and inorganic phosphate. Plays a role in regulating glucose sensing and insulin secretion of pancreatic β-cells. Modulates glycerol gluconeogenesis in liver.	Upregulated
UCA1	Urothelial Carcinoma-Associated 1<br><br>Long non-coding RNA (lncRNA) that has been reported to bind to micro RNAs in various cancers. Significantly enhanced in hypoxic cells, due to HIF-1α binding to its promotor sequence and causing its transcription	Upregulated
GJB3	Gap Junction Protein Beta 3<br><br>Member of the connexin gene family and when encoded the protein is a component of gap junctions. Alternative splicing results in multiple transcript variants encoding the same protein.	Upregulated

Supplemental Table S3. SLFN12 Upregulated Signature Genes

Gene	Function	Regulation Following LV-SLFN12
EEF1A2	Eukaryotic Translation Elongation Factor 1 Alpha 2<br><br>Enables translation elongation factor activity and acts upstream of or within positive regulation of apoptotic process and translational elongation. Located in cytoplasm.	Downregulated
NQO1	NADPH: Quinone Oxidoreductase 1<br><br>Cytoplasmic flavoenzyme which utilizes either NADH or NADPH as a substrate to reduce quinones into hydroquinones. Normal functions of NQO1 include superoxide scavenging, maintenance of antioxidant vitamins, and xenobiotic detoxification.	Downregulated
PAEP	Progesterone-Associated Endometrial Protein<br><br>Synthesized by human reproductive tract cells, especially in the secretory endometrium and decidua of early pregnancy. Inhibition of PAEP has been demonstrated to dramatically reduce colony formation and migration of malignant cells.	Downregulated
GJA1	Gap Junction Protein Alpha 1<br><br>Provides instructions for making a protein called connexin 43, which is one of 21 connexin proteins. Connexin 43 is found in many tissues such as the eyes, skin, bone, ears, heart, and brain, and it plays a role in their normal development and function.	Downregulated

Supplemental Table S4. SLFN12 Upregulated Signature Genes

A.

Gene 1	Gene 2	Treatment	P-Value	Degrees of Freedom	R^2 value	Coorelation coefficient
CALB2	EEF1A2	CPT	8.68E-13	72	0.5583	0.7279982
CALB2	NQO1	CPT	2.20E-16	57	0.7424	0.7843034
CALB2	FBP1	CPT	1.63E-14	69	0.6204	0.6220512
EEF1A2	FBP1	CPT	1.10E-14	69	0.6248	0.4415018
EEF1A2	NQO1	CPT	2.20E-16	58	0.7381	0.5668441
FBP1	NQO1	CPT	5.46E-12	59	0.6066	0.6974219
UCA1	GJB3	CPT	2.20E-16	59	0.797	0.4167834
UCA1	GJA1	CPT	0.000234	65	0.2556	-0.0806447
UCA1	PAEP	CPT	0.08069	63	0.1007	0.02720028
GJB3	PAEP	CPT	0.04863	51	0.1418	0.192267
GJA1	PAEP	CPT	0.2127	114	0.0385	-0.1919589
GJA1	GJB3	CPT	2.20E-16	55	0.7796	0.2565613

B.

Gene 1	Gene 2	Treatment	P-Value	Degrees of Freedom	R^2 value	Coorelation coefficient
UCA1	GJA1	ZA	3.58E-04	51	0.3009	0.1296646
UCA1	FBP1	ZA	5.84E-02	50	0.1375	0.2451742
UCA1	CALB2	ZA	3.50E-01	50	0.06298	0.3736543
GJA1	CALB2	ZA	4.06E-01	51	0.05494	-0.01723584
GJA1	FBP1	ZA	5.84E-02	50	0.1375	0.298026
FBP1	CALB2	ZA	5.50E-02	49	0.1424	0.1923173
GJB3	PAEP	ZA	5.38E-03	74	0.1565	0.2292605
GJB3	NQO1	ZA	0.03577	71	0.1128	0.5716207
GJB3	EEF1A2	ZA	0.00022	75	0.2272	0.4116978
PAEP	EEF1A2	ZA	0.000123	74	0.2423	0.2734653
NQO1	EEF1A2	ZA	0.000148	71	0.2471	0.1897076
NQO1	PAEP	ZA	6.52E-03	70	0.1597	0.1494987

C.

Gene 1	Gene 2	Treatment	P-Value	Degrees of Freedom	R^2 value	Coorelation coefficient
UCA1	GJB3	PAX	7.06E-01	83	0.01662	0.3396048
UCA1	PAEP	PAX	8.94E-03	35	0.2786	0.5502929
GJB3	PAEP	PAX	8.99E-03	36	0.2719	0.5915135
EEF1A2	GJA1	PAX	5.98E-01	72	0.0256	0.6491442

D.

Gene 1	Gene 2	Treatment	P-Value	Degrees of Freedom	R^2 value	Coorelation coefficient
CALB2	EEF1A2	Carboplatin	6.85E-01	67	0.0218	0.06275971
CALB2	NQO1	Carboplatin	1.43E-02	75	0.1307	-0.118167
CALB2	FBP1	Carboplatin	9.70E-03	50	0.2023	0.5068466
EEF1A2	FBP1	Carboplatin	9.70E-03	50	0.2023	0.09024113
EEF1A2	NQO1	Carboplatin	3.73E-02	68	0.1163	0.1766247
FBP1	NQO1	Carboplatin	5.14E-02	50	0.1424	0.12789
GJB3	PAEP	Carboplatin	4.79E-01	52	0.0461	0.2806388
GJB3	UCA1	Carboplatin	0.3353	51	0.06372	0.3047762
PAEP	UCA1	Carboplatin	0.5585	67	0.03017	0.2328784

Supplemental Table S5. Correlation Curve Analysis

Correlation data tables for (A) CPT, (B) ZA, (C) paclitaxel, and (D) carboplatin are shown.

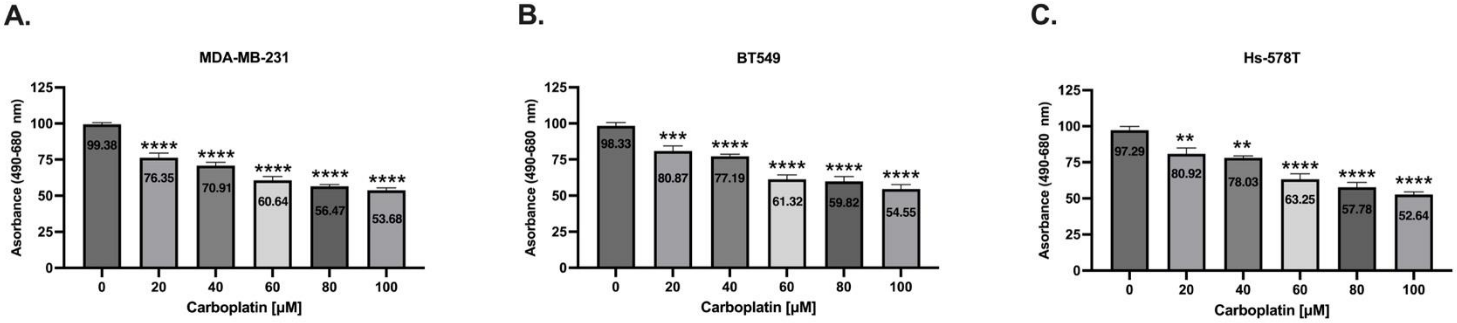

Supplemental Figure S1. Carboplatin Dose Response in TNBC.

Crystal violet assay showed a decrease in cell viability with treatments of carboplatin ranging from 0-100μM in (A) MDA-MB-231 (n=6, p<0.0001), (B) BT-549 (n=6, p<0.0001), and (C) Hs-578T (n=6, p<0.0001). Mean value is indicated in each bar. All error bars shown represent standard error of the mean. Asterisks denote significance between control and each value.

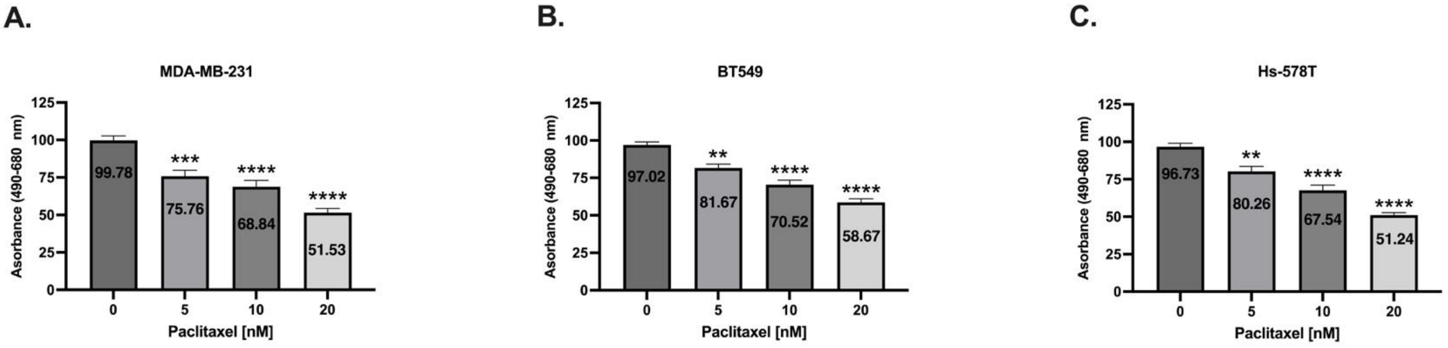

Supplemental Figure S2. Paclitaxel Dose Response in TNBC.

Crystal violet assay showed a decrease in cell viability with treatments of paclitaxel ranging from 0-20μM in (A) MDA-MB-231 (n=6, $p<0.0001$), (B) BT-549 (n=6, $p<0.0001$), and (C) Hs-578T (n=6, $p<0.0001$). Mean value is indicated in each bar. All error bars shown represent standard error of the mean. Asterisks denote significance between control and each value.

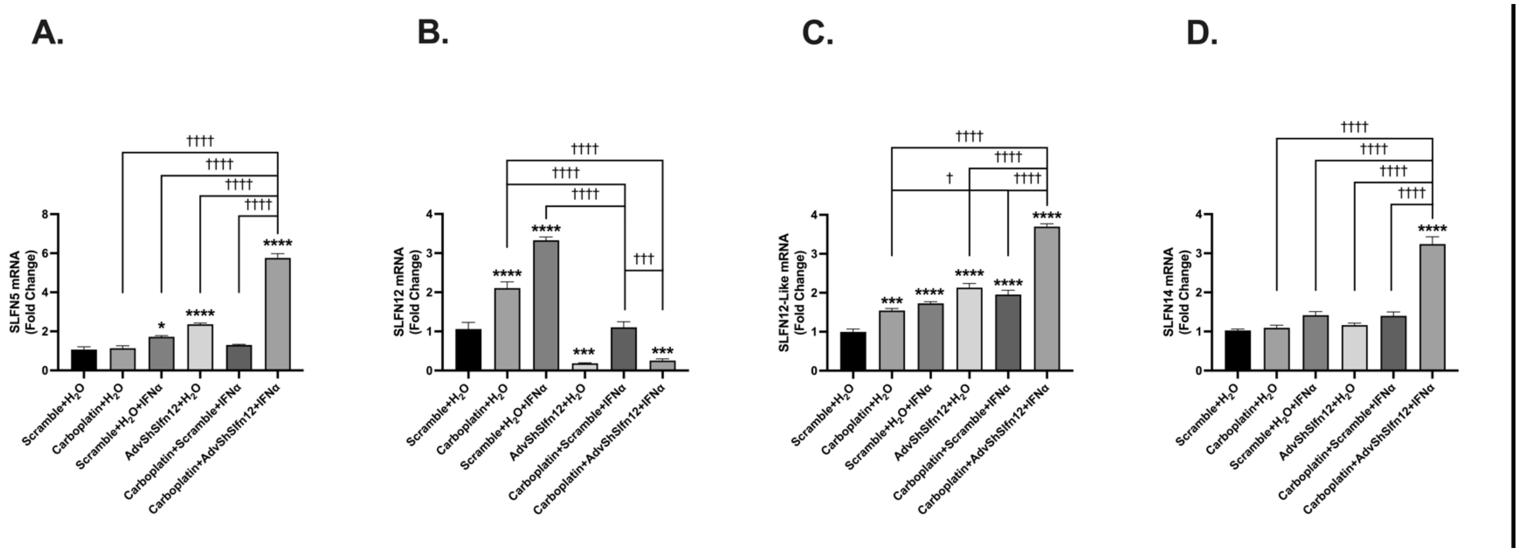

Supplemental Figure S3. SLFN family mRNA expression variably increases following carboplatin treatment paired with the loss of SLFN12 and IFN-α2 signaling in BT-549 cells. mRNA analysis performed by primer-probe RT-qPCR indicated that (A) SLFN5 (n=6, p<0.0001), (C) SLFN12-Like (n=6, p<0.0001), and (D) SLFN14 (n=6, p<0.0001) are induced by IFN-α2 treatment and significantly further induced with the loss of SLFN12 and carboplatin in BT-549 cells. RPLP0 was used as reference gene. All error bars shown represent standard error of the mean. Asterisks denote significance between control and each condition whereas crosses indicate significance between shown conditions. P value is for both asterisks and crosses; asterisks is for significance to Scramble + H_2O control and crosses are significance between experimental groups.

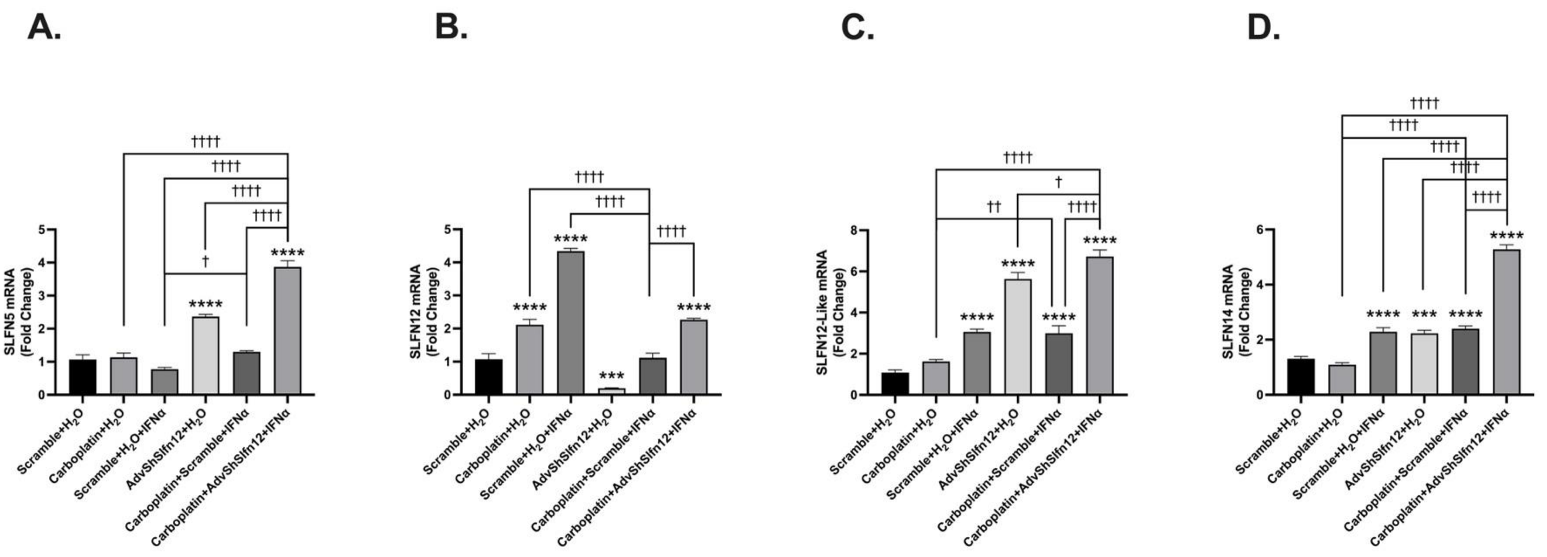

Supplemental Figure S4. SLFN family mRNA expression variably increases following carboplatin treatment paired with the loss of SLFN12 and IFN-α2 signaling in Hs-578T cells. mRNA analysis performed by primer-probe RT-qPCR indicated that (A) SLFN5 (n=6, p<0.0001), (C) SLFN12-Like (n=6, p<0.0001), and (D) SLFN14 (n=6, p<0.0001) are induced by IFN-α2 treatment and significantly further induced with the loss of SLFN12 and carboplatin in Hs-578T cells. RPLP0 used as reference gene. All error bars shown represent standard error of the mean. Asterisks denote significance between control and each condition whereas crosses indicate significance between shown conditions. P value is for both asterisks and crosses; asterisks is for significance to Scramble + H_2O control and crosses are significance between experimental groups.

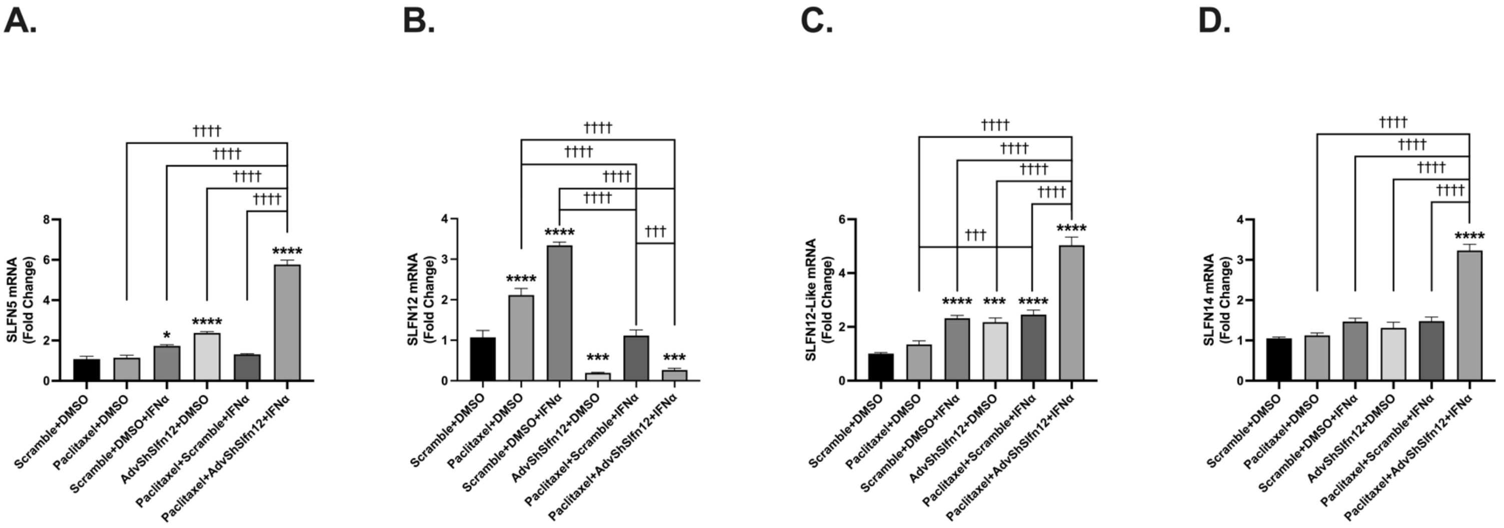

Supplemental Figure S5. SLFN family mRNA expression variably increases following paclitaxel treatment paired with the loss of SLFN12 and IFN-α2 signaling. mRNA analysis performed by primer-probe RT-qPCR indicated that (A) SLFN5 (n=6, p<0.0001), (C) SLFN12-Like (n=6, p<0.0001), and (D) SLFN14 (n=6, p<0.0001) are induced by IFN-α2 treatment and significantly further induced with the loss of SLFN12 and carboplatin in BT-549 cells. RPLP0 used as reference gene. All error bars shown represent standard error of the mean. Asterisks denote significance between control and each condition whereas crosses indicate significance between shown conditions. P value is for both asterisks and crosses; asterisks is for significance to Scramble + H_2O control and crosses are significance between experimental groups.

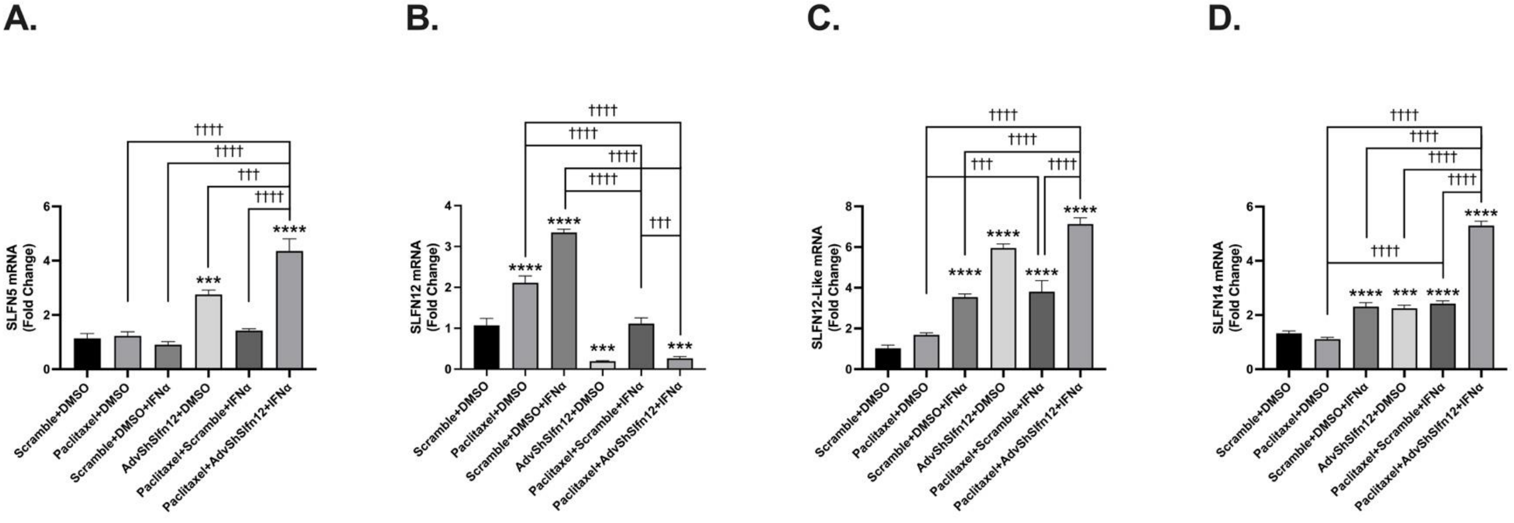

Supplemental Figure S6. SLFN family mRNA expression variably increases following paclitaxel treatment paired with the loss of SLFN12 and IFN-α2 signaling in Hs-578T cells. mRNA analysis performed by primer-probe RT-qPCR indicated that (A) SLFN5 (n=6, p<0.0001), (C) SLFN12-Like (n=6, p<0.0001), and (D) SLFN14 (n=6, p<0.0001) are induced by IFN-α2 treatment and significantly further induced with the loss of SLFN12 and carboplatin in Hs-578T cells. RPLP0 used as reference gene. All error bars shown represent standard error of the mean. Asterisks denote significance between control and each condition whereas crosses indicate significance between shown conditions. P value is for both asterisks and crosses; asterisks is for significance to Scramble + H_2O control and crosses are significance between experimental groups.
